# Association between Birth Weight and Serum Lipid Concentration in Premenopausal Japanese Women

**DOI:** 10.2188/jea.14.5

**Published:** 2005-03-18

**Authors:** Kaname Kanai, Chisato Nagata, Hiroyuki Shimizu

**Affiliations:** 1 Bureau of Social Welfare and Health, Tottori Prefectural Government; 2 Department of Public Health, Gifu University School of Medicine.

**Keywords:** birth weight, lipoproteins, HDL, premenopause, cholesterol, triglycerides

## Abstract

BACKGROUND: The relationships between birth weight and serum lipid concentrations in premenopausal Japanese women were not well identified and also diet and serum hormone status in these women would be considered.

METHODS: A total of 59 premenopausal Japanese women completed a self-administered questionnaire including basic demographic information, disease histories, and menstrual and reproductive histories. They were asked to obtain information on birth weight recorded in mother-and-baby notebook issued by municipality from their mother. Diet was assessed by daily diet records from day 2 through day 10 of the menstrual cycle. Blood sample was collected on day 11 of the cycle to measure serum lipid and hormone concentrations (total and high-density lipoprotein [HDL] cholesterols, triglyceride, estrone, estradiol, and sex hormone-binding globulin).

RESULTS: Birth weight was significantly correlated with HDL cholesterol (r=0.32, p=0.03), but no with total cholesterol and triglyceride after controlling for age. Neither estrogen nor sex hormone-binding globulin was significantly correlated with serum lipid concentrations after controlling for age and the number of days prior to the next menses. The correlation between birth weight and HDL cholesterol was not affected after additional adjustment for serum estrogen and intakes of protein, calcium, and iron.

CONCLUSION: These data suggest that intrauterine growth may be associated with lipid profile.

The association between low birth weight and high death rate from cardiovascular disease was reported in two previous studies for men and women,^1^^,^^2^ respectively. The studies suggested that cardiovascular disease originates impaired development in utero. However, these findings have remained to be poorly understood. Serum lipid concentrations are regarded as well-established predictors of the development of cardiovascular disease.^3^ It is worth studying relationship between birth weight and serum lipid profile. Few studies have evaluated this association. We examined this association in premenopausal women aged 20-46 years using baseline data from participants in a dietary soymilk-supplementation study. Collection of serum sex hormone levels in the intervention study enabled us to consider their possible confounding effects on the relationship among birth weight and serum lipid concentrations.

## METHODS

Study subjects consisted of 59 premenopausal women who participated in dietary-soymilk-intervention study. A total of 72 female students and teachers at a course given at a nurses’ training school were invited to the intervention study in 1997. Of these, 60 women participated in the study.^4^ After excluding one woman who did not provided information on birth weight, 59 women were studied in the present study. None of the women had a history of cancer, endogenous diseases, chronic liver diseases, and cardiovascular diseases. They were not also taking hormonal medications. The details of the subjects were described in the other report.^4^ The study was approved by the local review board, and each woman provided a written informed consent.

The subjects responded to a self-administered questionnaire providing basic demographic information, disease history, and menstrual and reproductive histories. In Japan, birth weight is recorded in mother-and-baby notebook issued by municipalities by law. The women were asked to obtain this information from their mothers who must keep the notebooks.

Exercise was assessed by asking the average hours per week spent performing various kinds of activities during the previous year. The details including its validity are described elsewhere.^5^

A fasting blood sample was collected on the morning of the day 11 of the menstrual cycle. The first day of the menstrual bleeding was defined as the day 1. Diet was assessed by a series of daily diet records from the day 2 through the day 10 of the cycle before the initiation of dietary intervention. Intake of macro- and micro-nutrients was estimated from the diet records using the Standard Table of Food Composition in Japan (4^th^ revised edition). Fatty acid composition was based on data published by Sasaki et al.^6^ The onset date of the following menstruation was reported by the subjects.

The blood samples were centrifuged and the serum was separated. The samples are stored at -80°C until assayed. The serum total cholesterol, high-density lipoprotein-cholesterol (HDL cholesterol), and triglyceride (TG), was determined by enzymatic assay using an Auto Analyzer (Hitachi, Tokyo, Japan). The reagent used was L-type Wako cholesterol purchased from Wako Junyaku, Osaka, Japan. HDL cholesterol was precipitated with heparin and calcium. Serum concentrations of estrone, estradiol, and sex hormone-binding globulin were determimed by radioimmunoassay using kits purchased from Eiken Chemical Co. Ltd.,(Tokyo), Diagnostic Products Corporation, Japan (Chiba), and Pharmacia & Upjohn Co., Ltd.,(Tokyo), respectively. The intra-assay coefficients of variation were 1.14% for total cholesterol, 1.36% for HDL cholesterol, 1.13% for TG, 7.4% for estrone, 2.5% for estradiol, and 7.8% for sex hormone-binding globulin.

We used Spearman correlation coefficients to evaluate the relations between birth weight and serum hormone levels to serum lipid concentrations. Partial correlations were calculated after controlling for potential confounders. By including the following variables in the models, we examined the potential confounding effects of age, body mass index (BMI), smoking status, status of student/teacher, parity, exercise habits, age at menarche, birth order, mother’s age at birth, and intake of alcohol and macro- and micronutrients. Some blood samples could not be collected on the day 11 of the cycle because of school holidays. Actual sampling dates ranged from the day 9 through the day 13 of the cycle. Even the samples were obtained at the same day according to the menstrual cycle, length of menstrual cycle varies among the subjects and this should affect estrogen concentrations. Therefore, to evaluate the relationships between serum estrogen and lipid concentrations, the number of days prior to the next menses was used for adjustment as covariates after categorizing it into <19, 19-23, and 24+ days.

## RESULTS

Descriptive characteristics and means for lipid and hormone levels are presented in Table 1. Of the women studied, 53 (90%) were nulliparous. Sixteen women reported that they were current smokers.

**Table 1.** Characteristics of the 59 subjects.

Variable	Mean	Standard deviation
Age (year)	27.1	7.4
Height (cm)	157.8	5.5
Weight (kg)	50.5	6.0
Body mass index (kg/m^2^)	20.4	2.1
Birth weight (g)	3,081	414
Age at menarche (year)	12.5	1.2
Exercise (METs • h/week)	16.6	23.8

Serum lipid concentrations		
Total cholesterol (mg/dL)	183.4	33.3
HDL cholesterol (mg/dL)	64.6	11.6
Triglyceride (mg/dL)	97.4	47.2

Serum hormone concentrations		
Estrone (pg/mL)	40.7	28.2
Estradiol (pg/mL)	860	75.0
Sex hormone-binding globulin (nmol/L)	63.9	23.6

Nutrient intake per day		
Energy (kcal)	1,664	237
Total fat (g)	55.1	9.8
Saturated fat (g)	16.7	3.4
Monounsaturated fat (g)	20.2	4.0
Polyunsaturated fat (g)	12.5	2.8
Protein (g)	58	9.6
Carbohydrate (g)	221	38
Cholesterol (mg)	276	80
Calcium (mg)	439	128
Iron (mg)	8	1.6
Ethanol (mL)	5.5	9.0

METs: metabolic equivalents

HDL cholesterol: high-density lipoprotein cholesterol

**Figure 1.**
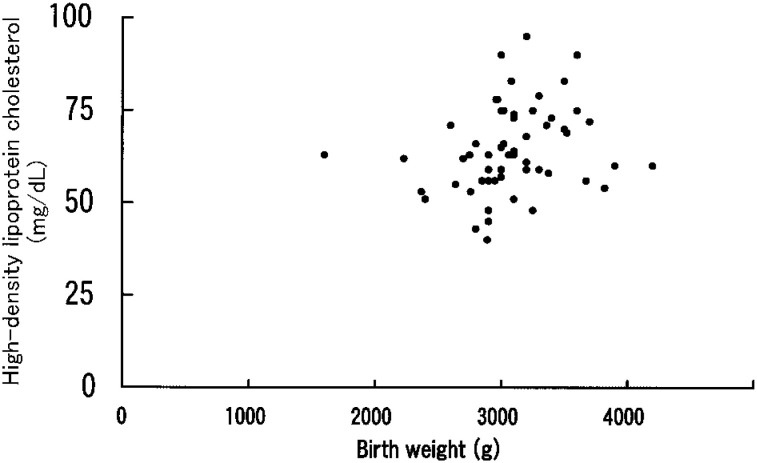
Relationship between birth weight and high-density lipoprotein cholesterol for the 59 premenoposal women.

Table 2 shows the correlations between birth weight and other non-dietary factors and lipid concentrations. Figure 1 shows the relationship of birth weight to HDL cholesterol. Birth weight was significantly correlated with HDL cholesterol (r = 0.32, p = 0.02), but not with total cholesterol and TG. Age at menarche was marginally significantly correlated with HDL cholesterol (r = 0.25, p = 0.06). The correlation between birth weight and HDL cholesterol remained significant after additional adjustment for age at menarche (r = 0.29, p = 0.03), while the correlation between age at menarche and HDL cholesterol was not significant after controlling for birth weight (r = 0.21, p = 0.12). Smoking was not associated with any lipid concentration; the means of total cholesterol concentrations were 182.9, 183.9, 191.0 mg/dL for never (n=41), current (n=16), and ex-smokers (n=2), respectively (p=0.95). The corresponding figures for HDL cholesterol and TG were 64.2, 63.6 and 80.5 (p=0.14) and 93.6, 110.4 and 69.5 mg/dl (p=0.34), respectively.

**Table 2.** Spearman correlation coefficients between birth weight and other non-dietary factors and serum lipid concentrations.

	Total cholesterol	HDL cholesterol	Triglyceride
Birth weight			
Crude	0.11	0.32*	-0.01
Adjusted for age	0.12	0.32*	0.02

Birth order			
Crude	-0.04	-0.08	-0.03
Adjusted for age	-0.01	-0.06	0.02

Mother’s age at birth			
Crude	-0.01	0.13	-0.16
Adjusted for age	-0.01	0.11	-0.12

Body mass index			
Crude	0.07	-0.03	0.07
Adjusted for age	0.06	-0.02	0.02

Age at menarche			
Crude	0.11	0.24	-0.11
Adjusted for age	0.11	0.25	-0.14

Alcohol			
Crude	-0.00	-0.06	0.22
Adjusted for age	-0.05	-0.05	0.15

Exercise			
Crude	0.06	-0.00	0.03
Adjusted for age	0.08	0.05	-0.09

*: p<0.05

HDL cholesterol: high-density lipoprotein cholesterol

Neither estrogen nor sex hormone-binding globulin was significantly correlated with serum lipid concentration after controlling for age and the number of days prior to the next menses (Table 3). Among the nutrient intakes, total protein, calcium, and iron were significantly correlated with HDL cholesterol after controlling for age and total energy (r = 0.26, p = 0.046, r = 0.26, p = 0.046, and r = 0.28, p = 0.03). The correlation between birth weight and HDL cholesterol was not affected after additional adjustment for intakes of total protein, calcium, iron, and serum estrone concentration (r = 0.31, p = 0.03).

**Table 3.** Spearman correlation coefficients between serum hormone and lipid concentration.

	Total cholesterol	HDL cholesterol	Triglyceride
Estrone			
Crude	0.10	0.18	0.05
Adjusted for age	0.09	0.20	0.02
Adjusted for age and number of days^†^	0.03	0.19	0.002

Estradiol			
Crude	-0.03	0.07	-0.03
Adjusted for age	-0.04	0.10	-0.13
Adjusted for age and number of days^†^	-0.19	0.06	-0.11

Sex hormone-binding hormone			
Crude	0.21	0.16	-0.04
Adjusted for age	0.20	0.19	-0.13
Adjusted for age and number of days^†^	0.20	0.18	-0.09
Adjusted for age, number of days^†^, and BMI	0.26	0.21	-0.08

^†^: number of days prior to the next menses

HDL cholesterol: high-density lipoprotein cholesterol

BMI: body mass index

## DISCUSSION

Birth weight was positively associated with HDL cholesterol in postmenopausal women from the Rancho Bernardo Study,^7^ but was unrelated to HDL cholesterol in men studied by Byberg et al.^8^ Birth weight was inversely associated with TG in boys and girls aged 7-11 years, although there was no significant association between birth weight and HDL cholesterol.^9^

Our finding of a positive correlation between birth weight and HDL cholesterol in premenopausal women is consistent with the result from the Rancho Bernado Study,^7^ in which the elevated HDL cholesterol was associated with high birth weight in postmenopausal women. This consistency suggests that sex hormone profile should not show substantial interrelations between birth weight and HDL cholesterol. We found that there was no significant confounding effect of serum estrogen and sex hormone-binding globulin on the association between birth weight and HDL cholesterol in premenopausal women. We speculate that maternal nutritional status can partially explain the link between birth weight and future lipid profile. Nutritional status during developmentally sensitive periods may cause the alternation of metabolism.

In the present study, we examined potential confounding effects of several variables including sex hormones, demographic factors, diet, smoking, and exercise. Sowers et al.^10^ reported that HDL cholesterol is related to three domains in premenopausal women: body measurements, sex hormone status, and carbohydrate metabolism. We could not include measurements of insulin resistance or glucose levels. We cannot deny a possibility that the association of low birth weight with low HDL cholesterol may be mediated by impaired carbohydrate metabolism.

Lipid concentrations were determined by a single measurement and this may have distorted the relationship between birth weight and HDL cholesterol. When we reanalyzed an association between birth weight and HDL cholesterol after 2 months of soymilk supplementation, this association was still significant (r = 0.38, p = 0.003) after controlling for age and group status. However, blood was drawn again on the day 11 of the menstrual cycle. As lipoprotein concentrations have been reported to fluctuate by phase of the menstrual cycle, it had been desirable that lipoprotein concentrations were measured repeatedly at different points in time. Lack of significant positive association between BMI and TG in our study may be due to the inclusion of few obese women (at most 25.6 of BMI). However, we cannot deny a possibility of effects due to the measurement error. Cyclic fluctuations in food intake occur in women across the menstrual cycle, with a periovulatory nadir and peak in the luteal phase. Diet record kept from the day 2 through the day 10, may have not been sensitive enough to reflect the interindividual variation in diet.
